# Posterior Protrusion Measures (PPM) as an Innovative Index in Classifying Plain Lateral Radiograph Images of Pertrochanteric Fracture Using the Revised AO Foundation/Orthopaedic Trauma Association (AO/OTA) Classification

**DOI:** 10.7759/cureus.32898

**Published:** 2022-12-24

**Authors:** Mitsuaki Noda, Shunsuke Takahara, Atsuyuki Inui, Keisuke Oe, Shin Osawa, Takehiko Matsushita

**Affiliations:** 1 Department of Orthopedics, Nishi Hospital, Kobe, JPN; 2 Department of Orthopedics, Hyogo Prefectural Kakogawa Medical Center, Kakogawa, JPN; 3 Department of Orthopedic Surgery, Kobe University Graduate School of Medicine, Kobe, JPN; 4 Department of Orthopedics, Kobe University Graduate School of Medicine, Kobe, JPN; 5 Department of Orthopedics, Nobuhara Hospital, Tatsuno, JPN

**Keywords:** plain radiograph, posterior protrusion measures, ao/ota classification, lateral view, pertrochanteric fracture

## Abstract

Introduction

The absence of a precise fracture classification system that classifies pertrochanteric fractures into either stable or unstable contributes to a burden on healthcare and has several major implications. We propose an innovative graphical index, which we refer to as posterior protrusion measures (PPM), using plain lateral view radiograph images for the revised AO Foundation (Arbeitsgemeinschaft für Osteosynthesesfragen)/Orthopedic Trauma Association (AO/OTA) classiﬁcation system. This study aims to: (i) introduce the use of PPM for classifying fractures into stable or unstable under the revised AO/OTA classification system and set the threshold numeric value, (ii) elucidate the reproducibility of inter and intra-observer agreement, and investigate the consistency of fracture classification using PPM versus computed tomography (CT) scan images.

Materials and methods

Out of 146 patients identified from the database, a total of 126 patients were enrolled in the study. Pertrochanteric fractures were classified as either stable or unstable. Three surgeons were assigned for PPM determination. Regarding the demographical data, the chi-square test was used to assess the significance of each parameter on a categorical scale between the two groups. The independent sample t-test or the Mann-Whitney U test was used to compare the two independent groups. Interclass correlation coefficient (ICC) values for continuous variables and kappa values (κ) for categorical variables were calculated to assess inter-observer and intra-observer agreement. Receiver-operating characteristic (ROC) analysis was used to determine optimal cut-off points of PPM to predict consistency between separate fracture classification groups, one using PPM values with a threshold derived from plain radiograph images, and the other using CT scan images.

Results

Among a total of 126 pertrochanteric fractures, the A1 (stable) group consisted of 39 patients (10 males, 29 females), whereas the A2 (unstable) group consisted of 87 patients (14 males, 73 females) (not significant, NS). Intraclass correlation coefficient (ICC) values of PPM for the inter-observer agreement were 0.796 (0.723-0.852), 0.664 (0.554-0.751), and 0.702 (0.601-0.781) at first examination and 0.729 (0.635-0.801) at the second. The intra-observer agreement was 0.869 (0.819-0.906) and 0.603 (0.480-0.703).

We examined for consistency of fracture classification group of PPM values with a threshold of 0.4 (A1<0.4, A2=0.4 or more) and CT-based group. For the first examination, there was mostly “moderate” agreement in fracture classification (stable or unstable) between plain radiograph and CT scan images, κ (95%CI): 0.427 (0.266-0.588), 0.493 (0.335-0.651), and 0.359 (0.176-0.544), and for the second, 0.418 (0.251-0.585), and 0.451 (0.284-0.620), respectively.

Conclusion

We propose a supplementary tool, namely PPM that allows for possible alternative classification of pertrochanteric fractures into A1 (stable) and A2 (unstable) using plain radiograph images under the revised AO/OTA classification system. In this study, a PPM threshold value of 0.4 demonstrated a moderate inter- and intra-observer agreement. It is noteworthy to mention that there was a satisfactory consistency of fracture classification using PPM derived from plain radiograph images when compared to classification using CT scan images. In addition, the PPM method provides a numerical score.

## Introduction

The rising incidence of femoral pertrochanteric fractures in the elderly population globally is a burden on healthcare systems, especially in developed countries [1]. The absence of a precise fracture classification system contributes to this situation and has several major implications. First, the existing guideline recommends dynamic hip screwing (DHS) for pertrochanteric fractures classified as stable, and using intra-medullary nails (IMN) or a modified DHS for unstable fractures [2-4]. With the results of the meta-analysis of unstable dislocation fractures, the proper use of implants, using IM nails rather than DHS, would enhance functional outcomes [5,6]. Second, from a clinical standpoint, a major postoperative complication such as implant breakage would likely occur in unstable fractures [7]. Third, epidemiological studies that correctly classified stable and unstable pertrochanteric fractures depicted an accurate picture of the trauma [8,9]. Considering these points, it is important to classify pertrochanteric fractures accurately into either stable or unstable fractures. Moreover, a classification system has to have a significant degree of reproducibility for it to be adapted for global use.

Among the various classification systems available worldwide, the revised AO Foundation (Arbeitsgemeinschaft für Osteosynthesesfragen)/Orthopedic Trauma Association (AO/OTA) classiﬁcation, introduced in 2018 [10], demonstrated a better reproducibility than the Evans-Jensen, Boyd, Grifﬁn, and even the original AO/OTA classiﬁcation system [11]. However, the revised AO/OTA classification system uses plain radiograph images and is limited by inter-observer reliability [12,13]. There is a need to improve the reliability of this revised system using better imaging techniques. Whereas, computed tomography (CT) scan images were reported to increase the reproducibility of pertrochanteric fracture classification using the revised AO/OTA classification system due to the fact that CT scan images can distinctively illustrate the complex morphology of posterior intertrochanteric region [14-16]. However, performing CT scans routinely in elderly patients with femoral trauma injuries may be difficult in an ordinary clinical setting due to the accessibility and cost of CT scans. Another major drawback of using CT scans is the added exposure of aged patients to ionizing radiation. For these reasons, a reproducible method using plain radiograph images should be applied for the classification of pertrochanteric fractures as either stable or unstable.

After reviewing multiple pairs of plain radiographs and three-dimensional (3D) CT scan images, we thought the sole critical index to differentiate A1 (stable) from A2 (unstable) fractures would locate at the posterior part of the greater trochanter, and concluded that the posterior protrusion in the plain lateral view is an important factor. Thus, we propose an innovative graphical index, which we refer to as posterior protrusion measures (PPM), using plain lateral view radiograph images. This method is simple and useful in providing orthopedic surgeons with a tool to classify pertrochanteric fractures. To our knowledge, the current literature does not have any mention of promoting this index using plain radiographic images for fracture classification. This study aims to: (i) introduce the use of PPM for classifying fractures into stable or unstable under the revised AO/OTA classification system and set the threshold numeric value, and (ii) elucidate the reproducibility of inter and intra-observer agreement, and investigate the consistency of fracture classification using PPM versus CT scan images. We hypothesize that the use of the PPM method would raise inter and intra-observer agreement and that a PPM-based classification would correlate favorably with CT scan evaluation.

## Materials and methods

Data source and collection

The data of patients diagnosed with pertrochanteric fractures from January 2019 to December 2020 were retrospectively collected from the surgical database of three hospitals in Western Japan: Nishi Hospital, Kasai Municipal Hospital, and Suzuran Hospital. Patients with both preoperative two-directional plain radiographs (anteroposterior and lateral views) and CT scan (3D-CT) images were included in the study. Exclusion criteria were as follows: (i) AO/OTA A3 type fractures, including those registered as subtrochanteric fractures, (ii) pathologic fractures and/or the presence of osteoarthritic changes, and (iii) previous history of ipsilateral hip fracture.

**Figure 1:**
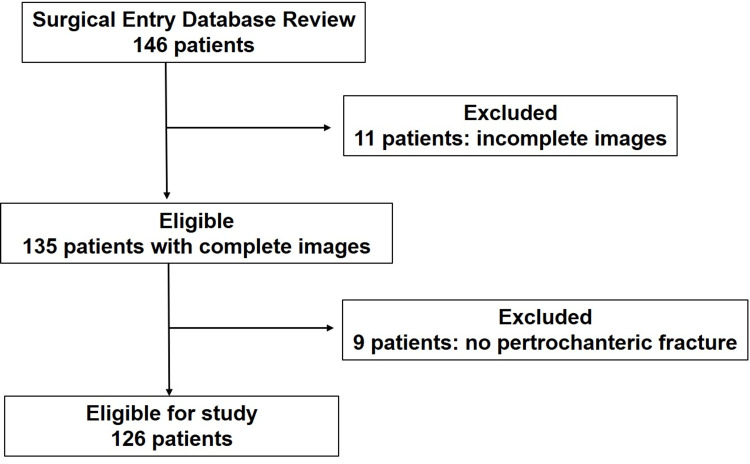
Flow diagram for patient selection, showing the patient inclusion and exclusion

Out of 146 patients identified from the database, 20 patients were excluded due to an incomplete set of plain radiographs and CT images (11 patients) and no pertrochanteric fracture (nine patients). A total of 126 patients were enrolled in the study (Figure 1). There were 24 male and 102 female patients. The mean age at the time of surgery was 87 years (50-99 years). Laterality of fracture was 70 left and 56 right.

Fracture classification

Pertrochanteric fractures were classified based on the revised AO/OTA classiﬁcation system using 3D-CT scans and plain radiograph images. Fractures were grouped as either A1 (stable) or A2 (unstable). Sub-classification was not necessary for the current study since only A1 and A2 classifications are useful in the actual clinical setting.

PPM using plain lateral radiograph images

**Figure 2:**
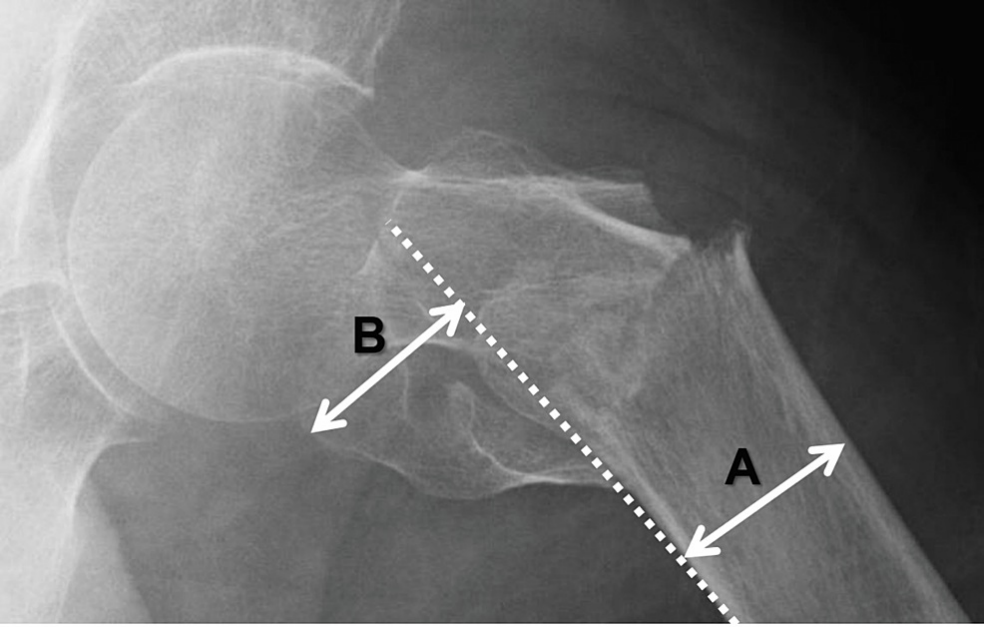
Posterior protrusion measure (PPM) PPM measures the degree of posterior displacement of the greater trochanter, and was used to classify pertrochanteric fractures into two groups: A1 (stable) and A2 (unstable). The formula for PPM is as follows: PPM = B/A A: femoral diameter; B: length of displaced greater trochanter; broken lines: a line along the posterior edge of the proximal femur

Two orthopedic surgeons (Observer 1 and Observer 2) and one resident (Observer 3) were assigned for PPM determination on a digital workstation, drawing lines and scaling lengths, and connecting the designated points, as shown in Figure 2. Each surgeon worked independently without any time restraint, magnifying and adjusting the brightness and contrast of the plain radiograph images for accurate identification of osseous structures. Two out of the three surgeons determined PPM values twice (first and second examination), with at least a one-month interval between each evaluation.

Method of Determining the PPM

(i) A line is drawn along the posterior edge of the proximal femur; (ii) The most posteriorly displaced point of the greater trochanteric fragment is marked and the perpendicular length between this point and the posterior edge line (‘displaced length’) is determined; (iii) A diameter of the proximal femur is measured; (iv) The PPM is determined and recorded, defined as the value of the ‘displaced length’ divided by the femoral diameter.

Statistical analysis

Statistical analysis was performed using EZR (Easy R) (Saitama Medical Center, Jichi Medical University, Saitama, Japan), a graphic user interface for R (The R Foundation for Statistical Computing, Vienna, Austria). A p-value under 0.05 was considered statistically significant.

Regarding the demographical data, the chi-square test was used to assess the significance of each parameter on a categorical scale between the two groups. Continuous variables were presented as mean ± standard deviation (SD), and the categorical variables were presented as numbers (n) and percentages (%). The normality of the data was assessed with tested using the Kolmogorov-Smirnov test. The independent sample t-test was used for the data in the normal distribution, and data outside the normal distribution, the Mann-Whitney U test was used to compare the two independent groups.

Interclass correlation coefficient (ICC) values for continuous variables and kappa values (κ) for categorical variables were calculated to assess inter-observer and intra-observer agreement. Interpretation for the coefficient values was designated as follows: poor agreement (less than 0.00); slight agreement (0.00-0.20); fair agreement (0.21-0.40); moderate agreement (0.41-0.60); substantial agreement (0.61-0.80); and almost perfect agreement (0.81-1.00) [17]. Receiver-operating characteristic (ROC) analysis was used to determine optimal cut-off points of PPM to predict the presence between separate groups. Area under curve (AUC) was defined as excellent (0.9-1), good (0.8-0.89), fair (0.7-0.79), poor (0.6-0.69), or fail/no discriminatory capacity (0.5-0.59) [18]. In this study, optimal cut-off points were defined by the Youden index when calculating AUCs. Youden’s index was calculated as maximum (sensitivity + specificity −1). Afterward, we examined the consistency of fracture classification using PPM derived from plain radiograph images when compared to classification using CT scan images. The variable PPM as an independent association factor was evaluated by univariate logistic regression analysis.

The confidence interval (CI) for the three observers using a sample size of 60 patients was also calculated, with a lower limit of 0.5 and an upper limit of 0.8 (α= 0.05) [14].

Ethics

This study was approved by the research ethics board of each of the three hospitals mentioned above, represented by the Nishi Hospital Ethical Committee (Approval number: 2021-1).

## Results

Comparison of demographic variables between A1 (stable) and A2 (unstable) fractures

A total of 126 pertrochanteric fractures were identified using CT scan images and plain radiographs in the revised AO/OTA classification system and were classified into two groups: A1 (stable) and A2 (unstable).

The A1 group consisted of 39 patients (10 males, 29 females), whereas the A2 group consisted of 87 patients (14 males, 73 females) (not significant, NS). The average age at the time of surgery in the A1 group was 87.0 ±6.4 years (range: 68-98 years), and 87.5 ±7.8 (range: 50-99 years) in the A2 group, the difference of which was not significant. Regarding the laterality of fracture site, there were 19 left and 20 right in the A1 group, and 51 left and 36 right in the A2 group (Table 1).

**Table 1:** Comparison of demographic variables between A1 (stable) and A2 (unstable) fractures ‡: P values for A1 and A2 group analyzed by gender and laterality of fracture site; †: Mean age and standard deviation; *: P-value of age groups NS: not significant

Variables	A1 (stable) N=39	A2 (unstable) N=87	p-value
Sex:	-	-	P=0.21‡
Male, n (%)	10 (25.6%)	14 (16.1%)	-
Female, n (%)	29 (74.4%)	73 (83.9%)	-
Age at surgery† (years)	87.0±6.4 (68~98)	87.5±7.7 (50~99)	P=0.40*
Laterality	-	-	NS
Left	19 (48.7%)	51 (58.6%)	-
Right	20 (51.3%)	36 (41.4%)	-

Evaluation of PPM inter- and intra-observer agreement

PMM ICC values were calculated to assess inter-observer agreement. The ICC was 0.796 (0.723-0.852) between Observer 1 and 2, 0.664 (0.554-0.751) between Observer 2 and 3, and 0.702 (0.601-0.781) between Observer 1 and 3 at the first examination. The ICC increased to 0.729 (0.635-0.801) between Observer 1 and 3 for the second examination. The intra-observer agreement was 0.869 (0.819-0.906) in Observer 1 and 0.603 (0.480-0.703) in Observer 3. All values reflected a moderate to substantial inter- and intra-observer agreement (Table 2).

**Table 2:** PPM inter- and intra-observer agreement showing the correlation coefficient Observers 1 and 2 are experienced orthopedic surgeons and Observer 3 is an orthopedic resident ICC: interclass correlation coefficient; PPM: posterior protrusion measures

Inter-Observer	ICC (range)	Intra-Observer	ICC (range)
1 vs 2	0.796(0.723-0.852)	1 vs 1	0.869(0.819-0.906)
2 vs 3	0.664(0.554-0.751)	3 vs 3	0.603(0.480-0.703)
1 vs 3 (1st examination)	0.702(0.601-0.781)	-	-
1 vs 3 (2nd examination)	0.729(0.635-0.801)	-	-

Evaluation of PPM values between A1 (stable) and A2 (unstable) groups

All PPM values were significantly lower in the A1 group compared to the A2 group, in both the first and second examinations (p<0.001). (Table 3). PPM value revealed A1/A2 in average±standard deviation, 0.21±0.20/ 0.53±0.27 in Observer 1, 0.24±0.18/ 0.59±0.28 in Observer 2, 0.38±0.21/ 0.67±0.29 in Observer 3 for the first examination, and 0.27±0.19/ 0.57±0.28 in Observer 1, 0.30±0.25/ 0.58±0.25 in Observer 3 for the second exam, respectively.

**Table 3:** PPM values in the A1 (stable) and A2 (unstable) groups Observers 1 and 2 are experienced orthopedic surgeons and Observer 3 is an orthopedic resident *: Independent sample T-test. **: Mann-Whitney U test PPM: posterior protrusion measures

Observer	A1 (stable) n=39	A2 (unstable) n=87	p-value
First examination	-	-	-
1	0.21±0.20	0.53±0.27	P<0.001*
2	0.24±0.18	0.59±0.28	P<0.001*
3	0.38±0.21	0.67±0.29	P<0.001*
Second examination	-	-	-
1	0.27±0.19	0.57±0.28	P<0.001*
3	0.30±0.25	0.58±0.25	P<0.001**

The cut-off PPM value was found 0.333 in Observer 1, 0.455 in Observer 2, 0.558 in Observer 3 for the first examination, and 0.442 in Observer 1, 0.393 in Observer 3 for the second exam, respectively, demonstrating both high sensitivity and high specificity (Table 4). A cut-off PPM value of 0.4 was set to represent the dichotomized classification of pertrochanteric fractures under the revised AO/OTA classification system.

**Table 4:** Cut-off PPM values for consistency of fracture classification AUC: area under curve. CI: confidence interval; PPM: posterior protrusion measures

Observer	AUC (range)	Cut-off	Sensitivity	Specificity
1st exam	-	-	-	-
1	0.838 (0.767-0.909)	0.333	80.5	77.4
2	0.864 (0.799-0.929)	0.455	70.1	89.7
3	0.797 (0.718-0.876)	0.558	60.9	87.2
2nd exam	-	-	-	-
1	0.819 (0.745-0.893)	0.442	70.1	82.1
3	0.795 (0.709-0.880)	0.393	82.8	66.7
Average		0.446		

Consistency of fracture classification by PPM: plain radiograph versus CT scan images

We examined for consistency of fracture classification using PPM values with a threshold of 0.4 (A1<0.4, A2=0.4 or more) derived from plain radiograph images and compared it to classification using CT scan images. For the first examination, there was a mostly moderate agreement in fracture classification (stable or unstable) between plain radiograph and CT scan images, κ (95%CI): 0.427 (0.266-0.588), 0.493(0.335-0.651), and 0.359 (0.176-0.544) among the three observers, respectively. The values (numbers (n)/ percentage (%)) for the exact estimate were 91/72.2, 96/76.2, and 92/73.0 in the three observers, respectively. The underestimate values for each observer were 8/6.3, 8/6.3, and 16/12.7, respectively. Overestimate values were 27/21.4, 22/17.5, and 18/14.3, respectively. Similarly, the second examination also demonstrated a “fair” to “moderate” agreement in fracture classification (stable or unstable) between plain radiograph and CT scan images, κ = 0.418(0.251-0.585), and 0.451(0.284-0.620) in Observers 1 and 3, respectively. The exact estimate values were 92/73.0, and 95/75.4, respectively. The underestimate values for each observer were 23/18.3, and 19/15.1, while overestimate values were 11/8.7, and 12/9.5, respectively (Table 5). Univariate logistic regression analysis for PPM showed the risk odds ratios of 7.5 in Observer 1, 8.1 in Observer 2, 3.9 in Observer 3 for the first examination, and 5.8 in Observer 1, 5.7 in Observer 3 for the second exam, respectively (Table 6).

**Table 5:** Consistency of fracture classification: PPM versus CT scan images Consistency of pertrochanteric fracture classification into A1 (stable) or A2 (unstable) between PPM-based evaluation versus CT scan images in three observers. Exact, under, and overestimates were calculated. Observers 1 and 2 are experienced orthopedic surgeons and Observer 3 is an orthopedic resident. CI: confidence interval; PPM: posterior protrusion measures

Observer	kappa coefficient (range)	Exact estimate n/%	Underestimate n/%	Overestimate n/%
1	0.427 (0.266~0.588)	91/72.2	8/6.3	27/21.4
2	0.493 (0.335-0.651)	96/76.2	8/6.3	22/17.5
3	0.359 (0.176-0.544)	92/73.0	16/12.7	18/14.3
1 (2nd examination)	0.418 (0.251-0.585)	92/73.0	23/18.3	11/8.7
3 (2nd examination)	0.452 (0.284-0.619)	95/75.4	19/15.1	12/9.5

**Table 6:** Univariate logistic regression analysis for PPM Observers 1 and 2 are experienced orthopedic surgeons and Observer 3 is an orthopedic resident. OR: odds ratio. CI: confidence interval; PPM: posterior protrusion measures

Observer	OR (range)	p-value
1st exam		
1	7.5 (3.6-15.7)	<0.001
2	8.1 (3.9-16.9)	<0.001
3	3.9 (2.4-6.6)	<0.001
2nd exam		
1	5.8 (3.1-11.0)	<0.001
3	5.7 (3.1-10.5)	<0.001

## Discussion

We propose a supplementary tool, namely PPM that allows for possible alternative classification of pertrochanteric fractures into A1 (stable) and A2 (unstable) using plain radiograph images under the revised AO/OTA classification system. In this study, a PPM threshold value of 0.4 demonstrated a substantial inter- and intra-observer agreement. It is noteworthy to mention that there was a moderate consistency of fracture classification using PPM derived from plain radiograph images when compared to classification using CT scan images. In addition, the PPM method provides a numerical score which allows for quantitative evaluation, whereas conventional classification is only qualitative.

Inter and intra-observer agreement comparing the PPM method with evaluation using lateral radiographs

In this study, both inter and intra-observer agreement was relatively accurate between all three observers (two experienced surgeons and one resident). On the contrary, other articles using two plain radiographs reported inferior scores, although the ICC values were not correctly compared with the kappa values. Iguchi et al. calculated the kappa value and reported that inter-observer agreements using plain radiographs were fairly reliable [19]. Chan et al. calculated a kappa value of 0.479, with intra- observer reliability increasing to 0.661 [12]. Klaber et al. described a moderate mean inter-observer agreement (k = 0.425) between six surgeons (three fellowship-trained hip surgeons and three orthopedic residents) [14]. Zarie et al. presented an inter-observer agreement value of 0.61 [13].

Rationale for the PPM method

We advocate that in using the revised AO/OTA classification system, the lateral view plain radiograph is more useful since the sole critical index to differentiate A1 (stable) from A2 (unstable) fractures would be the lateral wall thickness of the femur, cited as the distance 20 mm from a reference point and 3 cm below the innominate tubercle of the greater trochanter [14,20]. This anatomical spot identifies the posterior part of the greater trochanter. Thus, we presume that even a subtle displacement can be detected clearly in the lateral view, rationalizing the use of the PPM method. The anteroposterior view would hamper the detection of a posterior displacement fracture. This rationale is in line with 3D-CT images, depicting the posterior complex morphology in most pertrochanteric fractures [2,15].

A question may be asked regarding the value of taking two-directional plain radiograph images (anteroposterior and lateral) if only the lateral view would be sufficient for fracture classification. We surmised anteroposterior views have been given more emphasis than lateral views in fracture classification, which may lead to fracture misclassification. There are several articles describing the limited value of lateral views, although these articles used the traditional AO/OTA classification system, which gives less importance to posterior displacement [21,22]. Furui et al. acknowledged the importance of lateral views for postoperative reduction or rotation, but not for fracture classification [23]. In this study, we would like to give importance to the lateral view for the classification of pertrochanteric fractures using the revised AO/OTA classification system.

Does a high ICC score always lead to a reliable method?

Many articles argue that it is important to have excellent reliability in the classification of proximal femur fractures; however, we do not believe that this should be the sole decisive factor. Such a conclusion using the traditional AO/OTA classification system was reached by Cho et al. [2] involving 59 patients and four observers, and Wada et al. [24] analyzing 203 trochanteric fractures. Although we acknowledge the importance of inter- and intra-observer agreement, we believe that further analysis of consistency using more reliable graphical images such as CT is more crucial. In our separate study of classifying pertrochanteric fractures using plain radiographic images (without using PPM), there were fracture misclassifications among observers mainly due to the failure to recognize the posterior fragment. Such an error does not decrease the inter-observer reliability score when observers miss the same spots. In this current study, we demonstrate the consistency of PPM using plain radiographs versus CT scanning images as absolute values.

Strengths

The major strength of using the PPM method is its simplicity, applicability, and ease in aiding a surgeon’s treatment decision-making. Additionally, this method may be employed with various lateral views such as the Lauwenstein view, and cross-leg axial view [25]. Our preliminary saw-bone model, attached with a metal marked on the critical spot for lateral wall thickness (as designated by Hsu et al. [18]) passes over the extended line of the posterior margin of the proximal femur and could be applied in different radiographic lateral views. Another advantage of the PPM method is that it can be expressed numerically. PPM gives a quantitative measurement of pertrochanteric fractures, similar to Neer’s fracture classification where the proximal humerus includes a length index of 1cm as a displaced threshold [26]. The majority of conventional pertrochanteric fracture classification systems, such as AO/OTA, Evans, and Jensen, feature the use of a combination of osseous fragments.

Limitations

This study has several limitations. First, the number of observers was limited to three, and Observer 2 measured once, which was lesser compared to other studies [11,24]. However, PPM is fundamentally a geometric method that is less dependent on subjective judgment. If the observers are able to accurately identify the greater trochanteric outlines, the inter-observer agreement value will not be affected, leading to a high degree of reliability among the observers. Second, the current PPM study can only be applicable to a stable-unstable binominal classification. The complete five-class classification is scarcely used in the clinical setting; hence, the two groups (stable and unstable) are sufficient. Third, surgeons may be unaccustomed to measuring lengths, which is not required in ordinary fracture classification systems; however, PPM measurement and calculation require minimal practice. Fourth, inexperienced residents may be confused in fractures with an overlap of the ischial tuberosity and greater trochanter in the lateral view. Senior surgeons are encouraged to train them, and only a short period of teaching is sufficient.

Future direction

We would like to suggest a careful analysis of both the anteroposterior and lateral radiograph views. Close attention must be given not only to the anteroposterior view but also to the lateral view, focusing on the posterior osseous structures. This PPM method may be used to quantify the degree of femoral fracture displacement. In the past, the lateral view has been placed less weight, but we believe that this image is essential, especially when using the revised AO/OTA classification system. In addition, a slightly displaced fracture fragment often retains soft tissue stability, while the substantial displacement of fragments due to massive damage of surrounding organs results in an unstable fracture. In the future, we should possess better methods and tools to analyze and measure the exact extent of displacement.

## Conclusions

This study presents PPM, a supplementary method for classifying pertrochanteric fractures into stable and unstable under the revised AO/OTA classification system. The PPM method utilizes lateral radiograph images that measure the degree of posterior displacement of the greater trochanter. There is a significant inter- and intra-observer agreement with the use of this method; hence, improved consistency in classifying fractures, even when compared with classification using CT scan images.

This innovative method is simple and useful in the daily clinical practice of orthopedic surgeons and significantly contributes to the pertrochanteric classification system.
